# Supplementary figures and images for: Preconditioned Mesenchymal Stromal Cell-Derived Extracellular Vesicles (EVs) Counteract Inflammaging
Source: Cells. 2022 Nov 21;11(22):3695. doi: 10.3390/cells11223695 (PMC9688039; doi:10.3390/cells11223695)

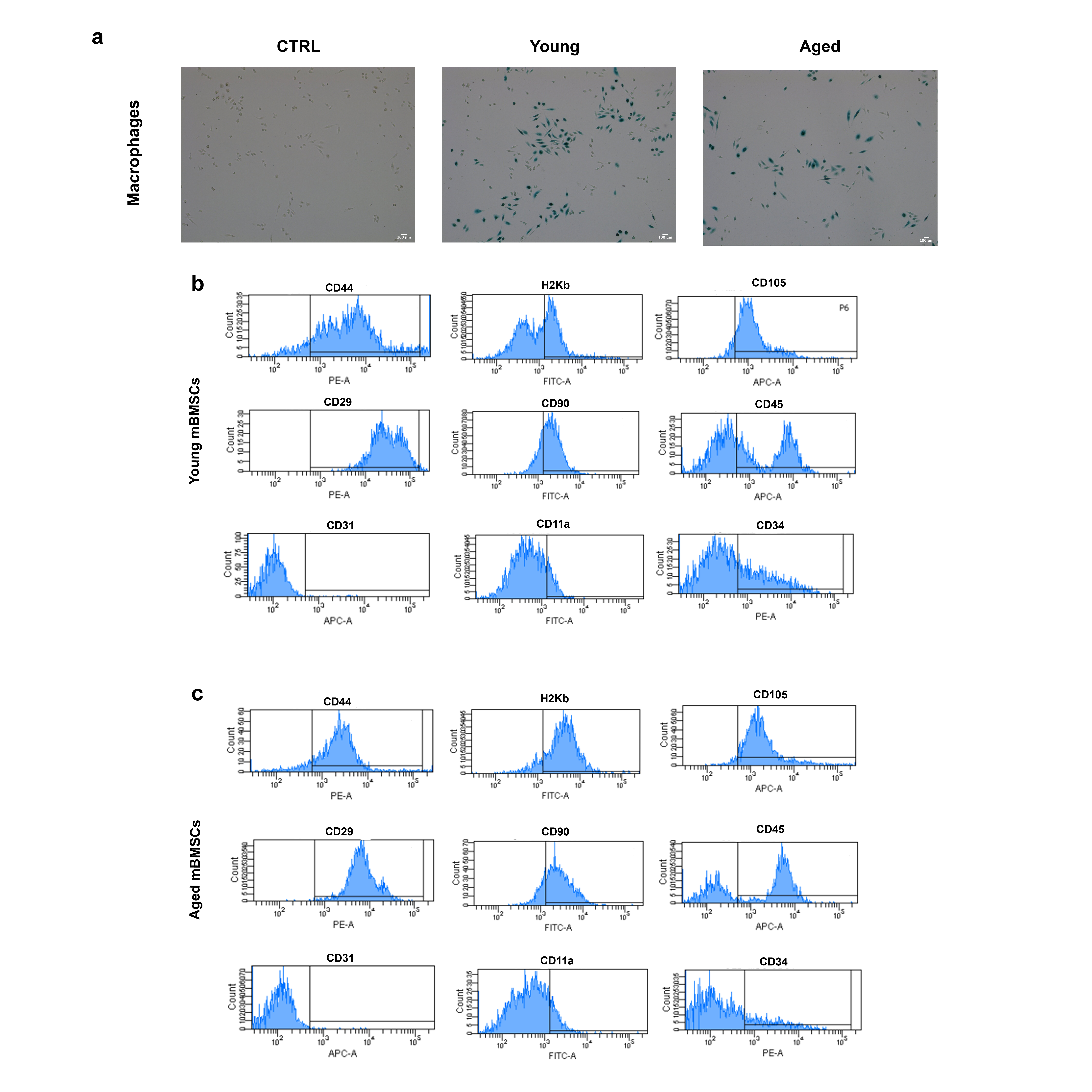

Supplement: Supplementary file 1 [file cells-11-03695-s001.zip › cells-1984140-supplementary.tif]
